# Mesenchymal Stem Cells for Liver Regeneration in Liver Failure: From Experimental Models to Clinical Trials

**DOI:** 10.1155/2019/3945672

**Published:** 2019-05-02

**Authors:** Maria P. de Miguel, I. Prieto, A. Moratilla, J. Arias, M. A. Aller

**Affiliations:** ^1^Cell Engineering Laboratory, La Paz Hospital Research Institute, IDiPAZ, Madrid, Spain; ^2^Department of General and Digestive Surgery, La Paz Hospital, Autonoma University of Madrid, Madrid, Spain; ^3^Department of Surgery, School of Medicine, Complutense University of Madrid, Madrid, Spain

## Abstract

The liver centralizes the systemic metabolism and thus controls and modulates the functions of the central and peripheral nervous systems, the immune system, and the endocrine system. In addition, the liver intervenes between the splanchnic and systemic venous circulation, determining an abdominal portal circulatory system. The liver displays a powerful regenerative potential that rebuilds the parenchyma after an injury. This regenerative mission is mainly carried out by resident liver cells. However, in many cases this regenerative capacity is insufficient and organ failure occurs. In normal livers, if the size of the liver is at least 30% of the original volume, hepatectomy can be performed safely. In cirrhotic livers, the threshold is 50% based on current practice and available data. Typically, portal vein embolization of the part of the liver that is going to be resected is employed to allow liver regeneration in two-stage liver resection after portal vein occlusion (PVO). However, hepatic resection often cannot be performed due to advanced disease progression or because it is not indicated in patients with cirrhosis. In such cases, liver transplantation is the only treatment possibility, and the need for transplantation is the common outcome of progressive liver disease. It is the only effective treatment and has high survival rates of 83% after the first year. However, donated organs are becoming less available, and mortality and the waiting lists have increased, leading to the initiation of living donor liver transplantations. This type of transplant has overall complications of 38%. In order to improve the treatment of hepatic injury, much research has been devoted to stem cells, in particular mesenchymal stem cells (MSCs), to promote liver regeneration. In this review, we will focus on the advances made using MSCs in animal models, human patients, ongoing clinical trials, and new strategies using 3D organoids.

## 1. Introduction

The liver has two functional characteristics that are fundamental to the maintenance of the organism's homeostasis. First, it centralizes the systemic metabolism and thus controls and modulates the functions of the central and peripheral nervous systems, the immune system, and the endocrine system. Hence, liver failure can cause encephalopathy, immunosuppression, and diabetes, respectively. Second, it intervenes between the splanchnic and systemic venous circulation, determining an abdominal portal circulatory system. For this reason, hepatic pathology can be the cause of portal vein flow obstruction with hypertension in the splanchnic venous circulation and development of portosystemic collateral circulation [1].

When the liver suffers an injury, either by viruses (hepatitis A, B, or C), toxic substances (alcohol), or immune (primary biliary cholangitis), metabolic (nonalcoholic fatty liver disease (NAFLD)), or tumoral (hepatocarcinoma) diseases, it displays a great capacity for regeneration [2].

## 2. Liver Failure and Regeneration from Intrinsic cells

### 2.1. Liver Failure Types

Liver failure is the consequence of a pathological progression that begins with hepatic parenchymal dysfunction and continues with progressive degrees of insufficiency until organ failure. At present, three types of liver failure are fully characterized:

- *Chronic Liver Failure*. This condition is hepatic cirrhosis in its final stages of evolution [3]. The evolution of cirrhosis depends mainly on its etiology. There are numerous classification systems to characterize the degree of liver failure and to predict the prognosis of cirrhotic patients. The most commonly used classification both for its simplicity and because it achieves an adequate evolutionary prediction is the so-called Child-Pugh-Turcotte score, which classifies three stages of cirrhosis, A, B, and C, the latter having the poorest prognosis. This score is based on severity of 3 impartial parameters (serum albumin level, serum bilirubin level, and prothrombin time) and 2 subjective parameters (ascites and encephalopathy)
- Also, to evaluate short-term mortality, a Model for End-Stage Liver Disease (MELD) has been instituted, based on the determination of creatinine and bilirubin, and it is an international normalized ratio. MELD is mainly used to prioritize treatment by liver transplant to patients with poorer prognoses [4–6]
- *Acute Liver Failure*. It is the sudden decompensation of hepatic function without previous hepatic pathology or with discrete hepatic insufficiency [7]. Patients show encephalopathy and coagulation alterations, although to classify the various types of acute liver failure, the timing of the appearance of the symptoms is used. Depending on whether the signs and symptoms appear at one week, between one and three weeks, or between three and twenty-six weeks is called hyperacute, acute, or subacute, respectively [7–9]
- *Acute-on-Chronic Liver Failure*. This condition is the functional liver failure characteristic of patients with cirrhosis who suffer from acute decompensation. It is a multifactorial hepatic pathology with ascites, hepatic encephalopathy, gastrointestinal hemorrhage, and/or bacterial infection [10]. These patients evolve rapidly in terms of multiorgan failure and high mortality rates. At present, it is considered that this syndrome is different from decompensated cirrhosis, given it has distinguishing characteristics, such as the fact that the systemic inflammatory response is more severe, although it is not caused by sepsis or by alcoholism

All of the abovementioned types of hepatic insufficiency would benefit from treatment by mesenchymal stem cell transplantation or by stimulating the intrinsic regenerative capacity of the hepatic parenchyma. In this sense, in chronic liver failure it appears more appropriate to test “in situ” regenerative therapies as there is a hepatic functional reserve susceptible to be activated. Thus, in chronic liver failure, a dedifferentiating stimulus of the remaining hepatocytes could constitute the establishment of regenerative niches of the parenchyma. In turn, in acute liver failure, it is predictable that the associated inflammatory response would hamper the effectiveness of intrinsic stem cell activation therapy. Conversely, the administration of mesenchymal stem cells or other cell therapy would be capable of counteracting this harmful stimulus by oxidative and enzymatic stresses, due to their anti-inflammatory and immunosuppressive properties, providing the necessary hepatocyte cellular support that substitutes the functional capacity which has been suppressed. Finally, in cases of acute-on-chronic liver failure, as in the case of acute liver failure, patients present a severe short-term prognosis, which limits their survival as well as the period of time necessary for cell replacement to take place effectively, so extrinsic MSC therapy and exquisite timing to be administered must be taken into account.

### 2.2. Hepatic Regeneration from Intrinsic Cells

The liver is a clearance organ and thus is subject to harmful substances, and it requires a powerful regenerative potential that rebuilds the injured parenchyma. This regenerative mission is mainly carried out by resident liver cells, either mature (hepatocytes and cholangiocytes) or with certain embryonic characteristics (hepatic stem/progenitor cells and biliary stem/progenitor cells) [11].

Hepatocytes and cholangiocytes have a great proliferative ability, and they stand out in terms of physiological hepatic turnover. In the liver lobule, the hepatocytes have various functional abilities depending on their location. While *β*-oxidation and gluconeogenesis are performed in the periportal hepatocytes (Rappaport zone 1), lipogenesis, glycogenolysis, and detoxification are carried out by the hepatocytes of Rappaport zone 3, corresponding to the vicinity of the central vein [12]. The proliferative capacity of hepatocytes is heterogeneous and depends both on their location and on the nature of the regenerative stimulus. Under physiological conditions, hepatocytes in zone 3 (centrilobular) are able to respond to a stimulus caused by toxic substances of intestinal origin proliferating rapidly [13]. On the other hand, the hepatocytes in zone 1 or periportal hepatocytes are capable of restoring the hepatic parenchyma that has suffered chronic aggression [14]. In addition, both subpopulations of hepatocytes can repopulate each other in situations of chronic toxic injuries or after hepatectomies [15].

At the same time, the hepatocytes have various pathways to reconstitute the liver mass depending on the type of injury. This characteristic has been demonstrated by performing various types of hepatectomies. Depending on the amount of hepatic parenchyma removed, such as 30%, 60%, and 80-90%, regeneration is mainly by hypertrophy, hyperplasia, or dedifferentiation in progenitor cells, respectively [13]. However, when the hepatic injury is accompanied by an inflammatory response, with hyperproduction of cytokines and chemokines, such as after episodes of ischemia/reperfusion injury, the increased expression of the transcription factor NF-*κ*B enhances hepatocyte proliferation [16]. Finally, the cholangiocytes are not only able to reconstitute the biliary epithelium, but in cases of severe hepatocyte failure their transdifferentiation towards hepatocytes occurs [17].

Cells with certain embryonic or immature characteristics involved in hepatobiliary regeneration are called stem/progenitor cells and are of two types: the hepatic stem/progenitor cells, with intrahepatic location, both in the canals of Hering and in the bile ductules, and the biliary stem/progenitor cells, which are located in the peribiliary glands of the large bile ducts and therefore are intra- and extrahepatic [18].

The hepatic stem/progenitor population exhibits bipotential differentiation capacity in both hepatocytes and cholangiocytes and expresses stem cell markers such as Sox 9, CD44, CD133, epithelial cell adhesion molecules (EpCAM), neural cell adhesion molecules (NCAM), and cholangiocyte (CK7, CK19) and hepatocyte (CK18) cytokeratins [19].

The activation of hepatic stem/progenitor cells depends on the cause of the injury and displays various phenotypes. In situations of hepatocyte injury (NAFLD, nonalcoholic steatohepatitis, cirrhosis, acute hepatitis, or cholangiopathies), an intermediate phenotype between stem and mature hepatocytes, so-called intermediate hepatocytes, is induced [20, 21]. However, when the lesion is biliary (biliary atresia, primary sclerosing cholangitis, or cholangiocarcinoma), the phenotype expressed by the hepatic stem/progenitor cells is biliary, with a proliferation of cells that express biliary traits and stem cell neuroendocrine markers [19–22]. In both cases, the activation of the hepatic stem/progenitor cells into the canals of Hering and bile ductules causes a ductular reaction, which participate in, among others, inflammatory mediators produced by hepatic stellate cells, portal myofibroblasts, and Kupffer cells [19].

**Figure 1:**
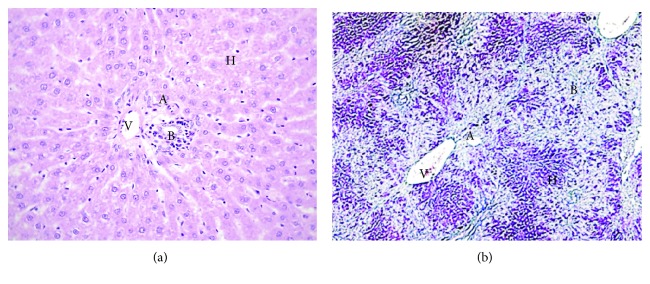
Histological images of a normal rat (a) and long-term cholestatic (b) liver parenchyma. Note the severe epithelial bile cell proliferation associated with fibrosis and hepatocyte death by necrosis and apoptosis in (b). V: portal vein, A: hepatic artery, B: biliary duct, and H: hepatocytes.

One of the consequences of the ductular reaction of hepatic stem/progenitor cells is the production of cirrhotic regeneration nodules, which do not possess the functional capacity of the hepatic lobule. These nodules are surrounded by fibrous tracts and cause portal hypertension with the development of collateral portosystemic circulation, both extra- (esophageal varices) and intrahepatic [4] (Figure 1).

The biliary stem/progenitor cells can differentiate into cholangiocytes, hepatocytes, and pancreatic islets [23, 24]. A subpopulation of these multipotent cells expresses Oct4, Sox2, and Nanog, which are markers of pluripotent stem cells [25, 26]. In hepatobiliary diseases, proliferation of the biliary stem/progenitor cells in the peribiliary glands causes hyperplasia. In particular, in primary sclerosing cholangitis, the remodeling of the peribiliary glands is associated with a chronic inflammatory response of the bile duct with the production of fibrosis. In its evolution, this chronic inflammatory process causes duct wall thickening and finally malignant degeneration with production of cholangiocarcinoma [27]. Both in this chronic inflammatory biliary pathology and in the biliary atresia, the peribiliary glands induce the production of Hedgehog pathway ligands involved in the epithelial-mesenchymal transition. This process enhances biliary fibrogenesis and consequently the production of stenotic lesions [27, 28].

Peribiliary gland vascularization originates from branches of the hepatic artery, and for this reason, in the case of hepatic arterial ischemia, they suffer from hypoxia with subsequent oxidative stress which, in turn, activates NF-*κ*B, causing inflammation [29]. This pathophysiological response has been observed in livers that have been transplanted orthotopically. In these cases, the deficient arterial or the excessive ischemia time would prevent the correct arterial revascularization of the bile duct and, consequently, the population of the biliary stem/progenitor cells would be activated in the peribiliary glands with a pathological reaction that leads to the development of nonanastomotic bile duct structures and cholestasis [30].

### 2.3. Liver Pathology and Inflammatory-Related Dedifferentiation

**Figure 2:**
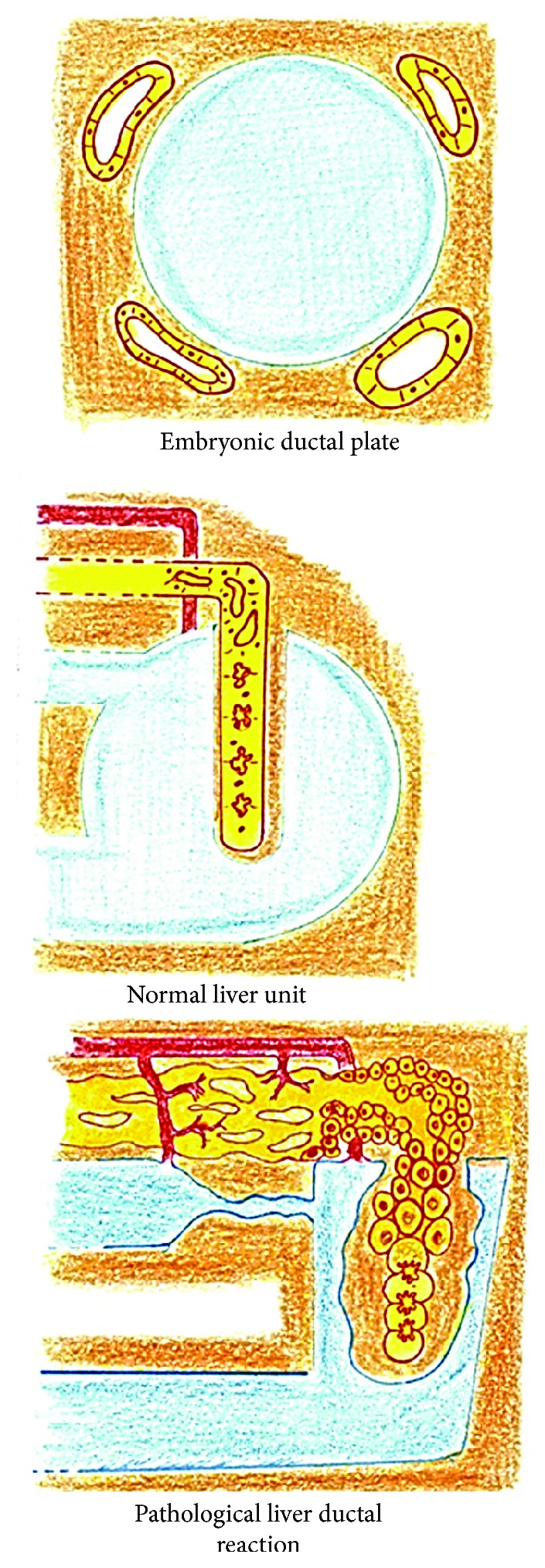
In the liver, the ductular reactions (bottom) could adopt ductal plate configurations (superior). In addition, the normal hepatic structure, represented by a functional hepatic unit (middle), is also based on the ductal plate configuration.

Inflammatory liver pathologies such as cholestatic diseases and benign and malignant tumors induce a dedifferentiation process in which structures that are common in its embryonic development are created and are histologically characterized by a massively increased number of bile duct structures [31]. The ductular reactions, as termed by Popper [32], form the paradigm of the liver dedifferentiation process [31] (Figure 2).

Three types of ductular reactions are recognized: *type 1* is predominant in acute complete bile duct obstruction and represents one of the myriad interactions between inflammatory, stromal, and bile duct cells. Type 1 results from the proliferation of preexisting cholangiocytes, resulting in elongation, branching, and luminal widening of biliary tubes [31]. *Type 2* can be subdivided in two types: *type 2A*, mostly periportal, which has been interpreted as “*ductular metaplasia of hepatocytes*” and is most characteristically observed in chronic cholestatic conditions. In addition, the cholestatic hepatocytes activate hepatic stellate cells into a myofibroblastic phenotype responsible for increased production of connective tissue matrix [31]. *Type 2B*, mostly centrilobular, occurs in parenchymal hypoxic areas, i.e., centrilobular in liver lobules and centronodular in cirrhotic nodules. Long-standing ischemia and hypoxia, such as in venous outflow block, result in the development of progressive perisinusoidal and centrilobular fibrosis and a concomitant reduction in the size of the hepatocytes in the centrilobular zone (centrilobular ductular metaplasia) [31, 33, 34]. *Type 3* consists of the activation and proliferation of liver stem/progenitor cells, which appear as periportal ductular structures in the case of massive hepatocellular necrosis. In most cases of fulminant liver failure with an unfavorable inflammatory microenvironment and progressive fibrosis, the liver progenitor cells evolve into cholangiocytic differentiation with an insufficient increase in parenchymal mass and greater development of ductular structures and accompanying fibrosis [31, 33].

In essence, ductular reactions are characterized by the proliferation of reactive bile ducts and are secondary to liver injuries [31, 35, 36]. The origin of active cells during ductular reactions could involve cholangiocytes, hepatocytes, or hepatic progenitor cells [36]. In this sense, hepatocytes can transdifferentiate into cholangiocytes if there is severe biliary damage and cholangiocytes can transdifferentiate into hepatocytes in certain conditions of severe hepatocyte damage [36]. Most ductular reactions occur according to Desmet's theory, in the form of small ductal plates composed of a small central blood vessel (altered sinusoid or venule) surrounded by a small amount of mesenchyme derived from the original Disse space, and typically, a double layer of biliary-type epithelial cells lining a circular, nearly virtual luminal cleft between both layers [31].

## 3. Current Liver Failure Treatments

Posthepatectomy hepatic failure remains at 10% of cases; one of the most frequently used criteria to predict prognosis in clinical practice is the 50-50 criterion that combines with PT index < 50% and serum total bilirubin > 50 *μ*mol/L (>2.9 mg/DL) on the postoperative day (POD) 5 [37, 38]. In normal livers, if the size of the liver is at least 30% of the original volume, the hepatectomy can be performed safely. In cirrhotic livers, the threshold is 50% based on current practice and available data. Typically, portal vein embolization of the part of the liver that is going to be resected is employed to allow liver regeneration in two-stage liver resection after portal vein occlusion (PVO). This strategy is one of the best in terms of avoiding hepatic insufficiency and allowing hepatic regeneration [39]. However, hepatic resection often cannot be performed due to advanced disease progression or a lack of indication in patients with cirrhosis. In such cases, liver transplantation is the only possible treatment. It is the only effective treatment, and it has very high survival rates of 83% after the first year; however, donated organs are becoming less and less available. Mortality and waiting lists have increased; hence, the living donor liver transplantation procedure was initiated. Such transplantation has overall complications of 38%. Another ALPPS technique associating liver partition and vein portal ligation for staged hepatectomy has an insufficient percentage of regrowth of liver remnants [38, 40, 41].

## 4. Cell Therapy for Liver Failure with MSCs

**Figure 3:**
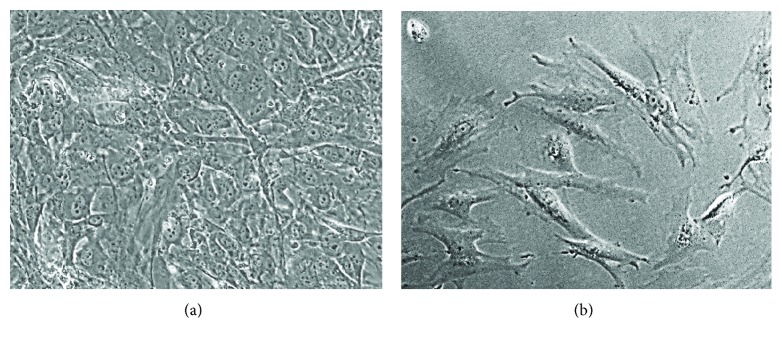
Mesenchymal stem cells in culture under phase-contrast microcopy. (a) Bone marrow-derived MSC. (b) Adipose tissue-derived MSC. Original magnification 200x.

In order to treat hepatic lesions, much research has been performed on stem cells, especially mesenchymal stem cells (MSCs), to promote liver regeneration after hepatic injury. MSCs have the ability to differentiate into hepatocytes and also to induce immunomodulatory and anti-inflammatory responses [42, 43]. MSCs can be obtained from multiple sources, including bone marrow, umbilical cord blood, and adipose tissue (Figure 3). They can stimulate liver regeneration after surgical resection, mainly by promoting hepatocyte proliferation, given that they secrete growth factors after liver injury and hepatic failure. Many studies have used MSCs to treat cirrhosis or to improve it, implying transdifferentiation into functional hepatocytes, and MSCs have also been shown to downregulate proinflammatory and fibrogenic cytokine activity, to stimulate hepatocellular proliferation, to promote collagen degradation by matrix metalloproteinases, and to reduce apoptosis of hepatocytes and therefore increase their proliferation.

Chemokines and cytokines secreted by MSCs might be effective in reducing inflammation and hepatocyte apoptosis in both acute and chronic liver injuries. MSCs have been shown to secrete epidermal growth factor (EGF), which promotes hepatocyte proliferation and function during liver regeneration [44]. MSCs have also been shown to reduce the proliferation of stellate cells and collagen type I synthesis through the secretion of TNF-*α*. Higashiyama et al. have suggested that MSCs mediate an antifibrotic effect through the expression of matrix metalloproteinase-9, which degrades the extracellular matrix [45]. No antifibrotic drugs are currently available; thus, MSC therapy could be promising for improving and preventing liver fibrosis [46].

### 4.1. Studies in Animal Models

Several animal models for both acute and chronic cirrhosis treatment with MSCs have shown benefits. Fang et al. [47] and later Zhu et al. [48] have shown reduced liver injury using undifferentiated MSCs in murine models of acute liver failure. Adipose-derived MSCs (AD-MSCs) show multipotency, and they can be differentiated into hepatocyte-like cells *in vitro* [49, 50]. These differentiated cells have shown expression of some hepatocyte markers, such as alpha-fetoprotein, GATA 4, cytokeratins 7 and 18, connexin 32, and E-cadherin, and production of proteins such as albumin, fibrinogen, cytochrome p450, and urea [49, 51–54]. *In vivo*, AD-MSCs were able to differentiate into hepatocytes and expressed albumin in immunodeficient mouse models, promoting hepatic integration [52, 54–56]. However, in a model of biliary fibrosis induced by bile duct ligation, engrafted bone marrow-derived MSCs (BM-MSCs) assumed an activated fibroblast or myofibroblast-like phenotype, aiding ductal fibrosis establishment [57]. These differences could be due in part to differences between BM-MSC and AD-MSC (Table 1). Treatment of acute injured liver in immunodeficient mice with predifferentiated AD-MSCs regenerated the liver [52]. Similar results have subsequently been obtained by Oyagi et al. [45, 53, 56, 58, 59].

Our studies on extrahepatic cholestasis-induced acute-on-chronic liver failure in rats demonstrated that isogenic hepatocyte-predifferentiated AD-MSCs intraparenchymally injected 2 weeks after the cholestasis were able to improve hepatic and extrahepatic complications [63]. The results demonstrated that rat AD-MSCs (isograft), predifferentiated or not, more effectively improved hepatic histological changes and ascites accumulation compared with human AD-MSCs (xenograft). In addition, predifferentiated rat cells have been shown to be more beneficial for treating liver fibrosis and for improving serum parameters of liver disease than undifferentiated cells [63].

In our model, isogenic transplantation of hepatocyte-predifferentiated AD-MSCs after microsurgical extrahepatic cholestasis reduced the hepatic and extrahepatic pathology secondary to long-term evolution, suggesting that AD-MSC-derived hepatocyte-like cells might be useful for the treatment of end-stage cholestatic liver disease. The direct incorporation of these cells into the fibrotic cholestatic liver could effectively improve the specialized hepatic metabolism and revert changes in the spleen and gonads that are a result of the inflammatory response [64, 65]. Based on our findings, we do not consider direct regeneration to be the major mechanism involved in the improvement of liver disease by AD-MSCs, given no proliferation or signs of hepatic regeneration specifically around the MSC injection site were observed. In accordance with our findings, the systemic therapeutic effects of MSC administration have been demonstrated in acute and chronic liver injury by indirect repair, that is, promoted by soluble factors secreted by the transplanted cells [48, 66–69].

In rats with obstructive cholestasis, portal fibroblasts are the first responders to liver injury [70, 71]; they proliferate and differentiate into myofibroblasts [72], which regulate cholangiocyte proliferation and interact, along with nonparenchymal cells, with fibrogenic stellate cells in order to stimulate their fibrogenic properties [70, 73]. In pathological conditions within a proinflammatory environment, hepatocyte stellate cells also play a principal role in liver fibrogenesis [74]. They differentiate into myofibroblasts that proliferate, migrate, and secrete excessive extracellular matrix proteins and proinflammatory and profibrogenic factors [72, 75]. Recently, the fibrogenic process has been shown to be reversible (for a review, see [76]). In our studies, fibrosis was reduced by MSC treatment, primarily by predifferentiated rat MSCs, suggesting that they produce soluble factors that counteract fibrogenesis cues in the liver parenchyma. In our experimental design, immunomodulatory properties of AD-MSCs also promoted a favorable environment for the stellate cells to maintain an anti-inflammatory phenotype, preventing immune cell-mediated liver injury [75, 77]. Accordingly, other groups have demonstrated that MSCs inhibit the immune response associated with acute liver failure [78] and have reported histological improvement, such as decreased fibrosis and inflammation in models of both acute and chronic liver injury [46, 66, 67, 69, 79–82].

MSCs might also exert their antifibrotic effects through the secretion of matrix metalloproteinases (MMP-9, MMP-13). These enzymes are normally upregulated during liver fibrosis in response to collagen accumulation, and an increase in their activity could allow a more efficient degradation of the extracellular matrix [83, 84]. Stem cells and VEGF-transfected MSCs transplanted into the portal vein were engrafted in the liver, and they significantly accelerated many parameters of the healing process following major hepatic resection. Okay et al. examined *in vitro* predifferentiated hepatocyte-like cells, which were then successfully used to treat liver fibrosis. In another study, the authors reported that MSCs that were predifferentiated into hepatocyte-like cells were more efficient for liver fibrosis prevention [83].

### 4.2. Cell Therapy with MSCs in Patients with Liver Failure

Clinical application of hepatocyte transplantation is prevented by the scarcity of donors, who are logically prioritized for whole organ transplant. Therefore, the use of pluripotent or multipotent cells differentiated toward hepatocytes has been the subject of intense research in patients (see [85], for a recent review). MSCs have several advantages over other cell types, such as their relatively simple acquisition and their strong proliferative capacity. In addition, MSCs can be injected repeatedly without loss of viability or function. In one study, autologous BM-MSCs were infused through the veins of four patients with decompensated cirrhosis. No adverse effects were observed, and End-Stage Liver Disease (MELD) score was improved in half of the patients. Kharaziha et al. [46] also reported improved liver function in patients with cirrhosis who were injected with autologous MSCs via the portal vein. Moreover, MSCs have been shown to improve liver function without severe adverse effects in the treatment of patients with liver cirrhosis of various causes, as has been shown in phase 1 studies [46, 81, 86].

There are currently 46 listed clinical trials involving MSC therapy for liver diseases, most focusing on cirrhosis (70%) but also on other acute liver diseases, such as liver failure and hepatitis [87]. The MSCs used in these trials are derived from bone marrow (51%), human umbilical cord (35%), adipose tissue (8%), and menstrual blood (2%) (Table 2). The major part of these cells were allogenic (65%), and the main route of administration was peripheral blood; however, many studies are also using interventional methods, via the hepatic artery or the portal vein. Most of these trials are registered in China (70%) and the Middle East (12%), but such studies are also taking place in India and Europe.

Most of these studies have not yet reported data. Three studies are not yet recruiting; one will attempt to use Stemchymal (commercial adipose-derived mesenchymal stem cells), and is estimated to be completed in 2020, and the other two will perform a classical MSC infusion via the peripheral vein. Eight of the studies are recruiting: five in China, two in Japan, and one in Spain. There is a long-term follow-up being performed of a completed clinical trial involving Livercellgram (autologous bone marrow-derived MSCs), enrolling by invitation. One of the trials using umbilical cord MSC transfusion in patients with severe liver cirrhosis has been suspended. Twenty-three of these trials have passed their completion date; however, their status has not been verified in more than 2 years. Ten studies have been completed; among them, we highlight those that are outstanding for the breadth of the research (phase 2 studies of end-stage liver failure) and the data provided.

In Kharaziha et al.'s group study [46], the study began with 20 patients with liver cirrhosis of various etiologies with no evidence of hepatocellular carcinoma; however, only 8 patients were reported at the end of the study. The MSCs were isolated from autologous bone marrow aspirate and were cultured over 2 months, leading to a loss of critically ill patients. Approximately 3 × 10^7^ to 5 × 10^7^ cells were injected through one of the main branches of the portal vein under ultrasound guidance (portal vein thrombosis occurred in two cases; thus, the injection was instead performed through the peripheral vein). Tracking of the MSCs after injection was not possible; therefore, the location in the body was not certain. Liver function was evaluated by MELD score, which improved in four patients. Regardless, the injection of MSCs was feasible, and all patients had a subjective improvement in quality of life; however, a higher number of patients with long-term follow-up and randomized controlled studies are necessary.

In another study [88], 25 patients with various cirrhosis etiologies were selected to undergo autologous BM-MSC transplantation. Due to end-stage disease complications and technical problems with the quality of the MSCs, only 12 of these patients completed the study. They received 1 × 10^6^ cells/kg via the peripheral vein, screening biochemical parameters monthly and performing a liver biopsy before and 6 months after transplantation. Eight of the patients showed improvement in the MELD score; fibrosis was the same before and after transplantation. Although injection via the peripheral vein is minimally invasive, the cell destination is unclear, and it is probable they did not reach the liver, a notion supported by the absence of differences between the liver biopsies in terms of liver tissue regeneration.

The study by Suk et al. [89] is a phase 2 clinical trial with 55 patients with alcoholic cirrhosis. They were randomized into a control group and an autologous BM-MSC group that received a hepatic arterial injection of 5 × 10^7^ cells 30 days after the aspiration or two injections 1 month and 2 months after the BM-MSC isolation. A first liver biopsy was performed before transplantation and at 6 months after the surgery, and a follow-up biopsy and blood study were performed, revealing improvement of the MELD score, fibrosis regression, and Child-Pugh score in the BM-MSC groups; however, no differences between the two-time BM-MSC and one-time BM-MSC transplantation were reported. Tracking the injected BM-MSCs was not possible, and the fibrosis reduction was not explained; thus, further studies are needed to demonstrate the effectiveness of mesenchymal stem cell therapy.

Amer et al. [83] showed improved MELD scores by predifferentiated BM-MSC administration. They randomized 40 patients with end-stage liver failure due to chronic hepatitis C into two groups of 20 patients: the first group received autologous bone marrow-derived MSCs previously transdifferentiated into hepatocyte-like cells *in vitro*; the second group received standard supportive treatment [83]. The patients receiving MSCs had significant improvement in Child-Pugh and MELD scores after 2 weeks, and they maintained this change for 6 months compared with controls. More recently, in a phase 2 trial, Zhang et al. [95] randomized (2 : 1) 46 patients with chronic hepatitis B receiving either three injections with 0.5 million/kg allogeneic umbilical cord-derived MSCs (*n* = 31) or saline solution (*n* = 15). Patients receiving MSC infusion had improved MELD scores and improved levels of ascites and fibrosis markers. Intraportal infusion appeared to be more efficient than via the peripheral route [83], and differentiation toward hepatocytes prior to infusion appeared not to increase MSC curative potential [90].

Similar results were reported by Peng et al. [91, 98]. Other studies, however, even from the same researchers, showed no benefit [92]. Also, it is not clear in patients whether MSCs diminish or contribute to fibrogenesis in the liver, and whether this is dependent on the route and the time frame of administration [39, 99, 100]; thus, more research is needed before MSC therapy as a mainstream treatment for liver failure can be established (for an outstanding and concise review, see Volarevic et al. [101]).

## 5. Future Approaches Using Tissue Engineering

New strategies for liver regeneration will take advantage of the progress in tissue engineering and the use of 3D scaffolds. Efforts have focused on *in vitro* generation of liver organoids using natural [102] and synthetic (hydrogels as in Skardal et al. [103]) materials, fluid flow [104], and 3D culture [103, 104] or 3D bioprinting [105–107]. Most studies focus on the development of liver organoids for liver disease modeling. In this regard, a pioneering study by Uygun et al. [108] was able to recellularize the architecture of a decellularized liver *in vitro* and more importantly demonstrate its viability on its own. Further, transplantation in rats maintained hepatocyte survival in the organoid. Hepatobiliary organoids able to survive *in vivo* have also been recently developed [109]. More recently, hepatic organoids with biliary structures have been generated [110].

With respect to treatment with organoids, remarkably Takebe et al. [111] generated a functional liver organoid *in vivo* by transplant of liver buds with vasculature generated *in vitro*. More recently, Nie et al. [112] claim to have improved the survival rate in acute liver failure mice transplanted with liver organoids generated from human cells (induced pluripotent stem cells, endothelial cells, and umbilical cord (MSC)) from a single donor. In this study, liver organoids were superior in hepatic capacity than umbilical cord-MSC. Bioartificial livers made form porcine liver organoids have reached the nonhuman primate stage [113], demonstrating increased survival for acute liver failure. However, not enough data has been yet generated to be able for comparison with MSC treatment.

## 6. Conclusions

In conclusion, despite the huge regenerative capacity of the liver after an injury, many diseases involving inflammation or advanced pathology require new strategies to promote liver regeneration *in vivo*. The use of mesenchymal stem cells is a valid option as demonstrated by many studies and ongoing clinical trials. The comparison of cell sources, administration route, and dosage, together with new strategies such as 3D-bioprinting, is an exciting and still unresolved area of research.

## Figures and Tables

**Table 1:** Differences in cell membrane CD expression and differentiation capacity between BM-MSC and AD-MSC. Data from [60–62].

Surface markers			Differentiation capacity		
	AD-MSC	BM-MSC		AD-MSC	BM-MSC
CD9	+	+	*Adipogenic efficiency*		
CD10	+	+	PPAR*γ*	High	High
CD11b	+	+	LPL	High	High
CD13	+	+	*Osteogenic efficiency*		
CD29	+	+	Osterix	Low	High
CD34	Unstable	−	Alk phosphatase	High	High
CD44	+	+	Osteocalcin	Low	High
CD45	−	−	*Chondrogenic efficiency*		
CD49d	+	−	Type II collagen	High	Low
CD54	+	Unstable	Aggrecan	Low	High
CD55	+	+	Type X collagen	High	Low
CD58	+	+	*Pancreatic efficiency*		
CD71	+	+	Insulin	Positive	ND
CD73	+	+	*Myogenic efficiency*		
CD90	+	+	Sarcomeric actin	Positive	ND
CD91	+	+	GATA4	Positive	ND
CD105	+	+	*Hepatic efficiency*		
CD106	+	+	Albumin	Positive	ND
CD140	−	+			
CD146	+	+			
CD166	−	+			

**Table 2:** Summary of clinical trials with MSC for liver failure.

Trial PI	Number of patients	Cell type	Cell number	Administration route	Disease
Kharaziha et al. [46]	8	BM-MSCs	3 × 10^7^ to 5 × 10^7^	Portal vein	Chronic liver failure
Amer et al. [83]	40	BM-MSCs	2 × 10^7^ cells	Intrasplenic vs. intrahepatic	End-stage liver failure
Kantarcıoğlu et al. [88]	12	BM-MSCs	1 × 10^6^ cells/kg	Peripheral vein	Liver cirrhosis
Suk et al. [89]	55	BM-MSCs	5 × 10^7^	Hepatic artery	Liver cirrhosis
El-Ansary et al. [90]	12	BM-MSCs	1 × 10^6^ cells	Intrasplenic vs. peripheral vein	Chronic liver failure
Peng et al. [91]	23	BM-MSCs	1 × 10^7^ cells	Hepatic artery	Liver failure
Mohamadnejad et al. [92]	25	BM-MSCs	1.95 × 10^8^ cells	Peripheral vein	Decompensated liver cirrhosis
Zhang et al. [93]	46	UC-MSCs	0.5 × 10^6^/kg	Peripheral vein	Decompensated liver cirrhosis
Yu et al. [94]	35	BM-MSCs	5 × 10^6^ cells	Peripheral vein	End-stage liver failure
Zhang et al. [95]	30	UC-MSCs	≥2 × 10^7^ cells	Hepatic artery	Decompensated liver cirrhosis
Liu et al. [96]	35	UC-MSCs	>5 × 10^7^ cells	Peripheral vein vs. hepatic artery	Acute-on-chronic liver failure
Sakai et al. [97]	4	AD-MSCs	3.3 × 10^5^ to 6.6 × 10^5^ cells/kg	Hepatic artery	Liver cirrhosis
